# Analysis of nationwide hemophilia care: A cohort study using two Japanese healthcare claims databases

**DOI:** 10.1002/hsr2.498

**Published:** 2022-01-27

**Authors:** Ei Kinai, Midori Ono, Akinori Oh, Mihoko Ota, Yasuo Myaguchi, Hitoshi Ueda

**Affiliations:** ^1^ Department of Laboratory Medicine Tokyo Medical University Tokyo Japan; ^2^ Japan Medical Office, Takeda Pharmaceutical Company Limited Tokyo Japan

**Keywords:** cohort study, disease management, factor VIII, hemophilia A

## Abstract

**Background and aims:**

In many developed countries, hemophilia care is provided by specialized centers which can offer standardized high‐quality care for patients and collect data for patient registries. However, in countries with less centralized provision of hemophilia care, registry data lacks accuracy and medical care is inconsistent among providers. Claims databases can be an alternative for obtaining nationwide data on hemophilia care, and we applied this approach to evaluate inequalities in hemophilia care in Japan.

**Methods:**

Medical records of hemophilia A patients were collected by a combination of ICD‐10 code (D66) and prescribed coagulation factors from two major Japanese claims databases (JMDC and Medical Data Vision [MDV]). Patient records with an anti‐inhibitor coagulant complex were excluded.

Based on the annual number of hemophilia A patients, medical facilities were categorized into specialized facilities (SP, ≥5 patients) and nonspecialized facilities (N‐SP, <5 patients). Patient age, comorbidities, diagnostic testing, prescribed drugs and their dosages were compared between facility types.

**Results:**

The JMDC and MDV databases included 274 and 1266 hemophilia A patients, respectively. In the MDV database, SP facilities prescribed extended half‐life factor VIII (FVIII) products for more patients (31.8% vs 24.3%) than N‐SP. The mean annual FVIII consumption per patient was higher in SP facilities (240 333 IU [international units] vs 210 334 IU), and the mean FVIII dosage was higher in SP facilities for all types of FVIII products. The proportion of patients who received diagnostic blood tests was higher in SP (75.7% vs 56.2%).

**Conclusion:**

The MDV database revealed disparities in hemophilia A care between SP and N‐SP facilities in types of FVIII products prescribed, FVIII consumption, and frequency of the relevant management such as blood tests. Claims databases can be an alternative for the assessment of nationwide hemophilia care patterns in countries without a well‐established registry.

## 1 INTRODUCTION

Hemophilia A is an X‐linked hereditary disorder that results from a deficiency or dysfunction of the factor VIII (FVIII) coagulation protein, causing recurrent joint and muscle bleeds and leading to progressive musculoskeletal damage.^1^ Estimates of the global prevalence for all severities is 17.1 cases per 100 000 males and 6.0 per 100 000 males for severe cases.^2^ In Japan, a survey conducted in 2018 reported an estimated 8751 cases of coagulation disorders, of which 5301 involved hemophilia A.^3^

According to current guidelines of the World Federation of Hemophilia, the standard of care for hemophilia A patients is treatment with FVIII concentrates^4^; both recombinant FVIII (rFVIII) or plasma‐derived FVIII (pdFVIII) products are available, and factor replacement therapy may be administered in an episodic or prophylactic manner.^4^ The efficacy of regular prophylactic rFVIII infusions for the prevention of joint damage and other hemorrhages is supported with high‐quality evidence from a randomized, open‐label study.^5^ Multiple standard half‐life (SHL) rFVIII products are now available, but they need intravenous administration as often as every other day.^6^ Protein conjugation, chemical modification or protein sequence modification has been used to develop extended half‐life (EHL) rFVIII products to satisfy patient expectations of less frequent injections.^7^, ^8^, ^9^, ^10^ Emicizumab—a bispecific antibody that replaces the function of missing activated FVIII thereby restoring hemostasis—is a novel, non‐factor treatment for hemophilia A.^11^ Emicizumab received licensing approval for treatment of hemophilia A with or without inhibitors in Japan in 2018.

In the majority of high‐income countries, patients with hemophilia A have access to specialized hemophilia care centers that offer a full range of options for management of bleeding disorders including prescription of coagulation factors, monitoring of hemostatic parameters, consultation on prophylaxis, medical management of joint health and information on new treatments and clinical trials.^12^ In a Japanese survey, 186 out of 290 hospitals provided care for fewer than five blood coagulation disorder patients annually.^3^ Although these data are not limited to hemophilia, they imply that many patients are receiving treatment in nonspecialized medical facilities. To expand on these findings, we sought to better understand the current patterns in the treatment and management of hemophilia A in Japan and conducted a retrospective claims‐based cohort study of two large patient databases. The specific objectives were to determine whether there are any differences in the provision of care for patients with hemophilia A between specialized and nonspecialized hospitals and clinics (defined as those treating ≥5 and <5 hemophilia A patients per year, respectively), with respect to the therapeutic agent selection and dose.

## 2 MATERIALS AND METHODS

### 2.1 Study design

**FIGURE 1:**
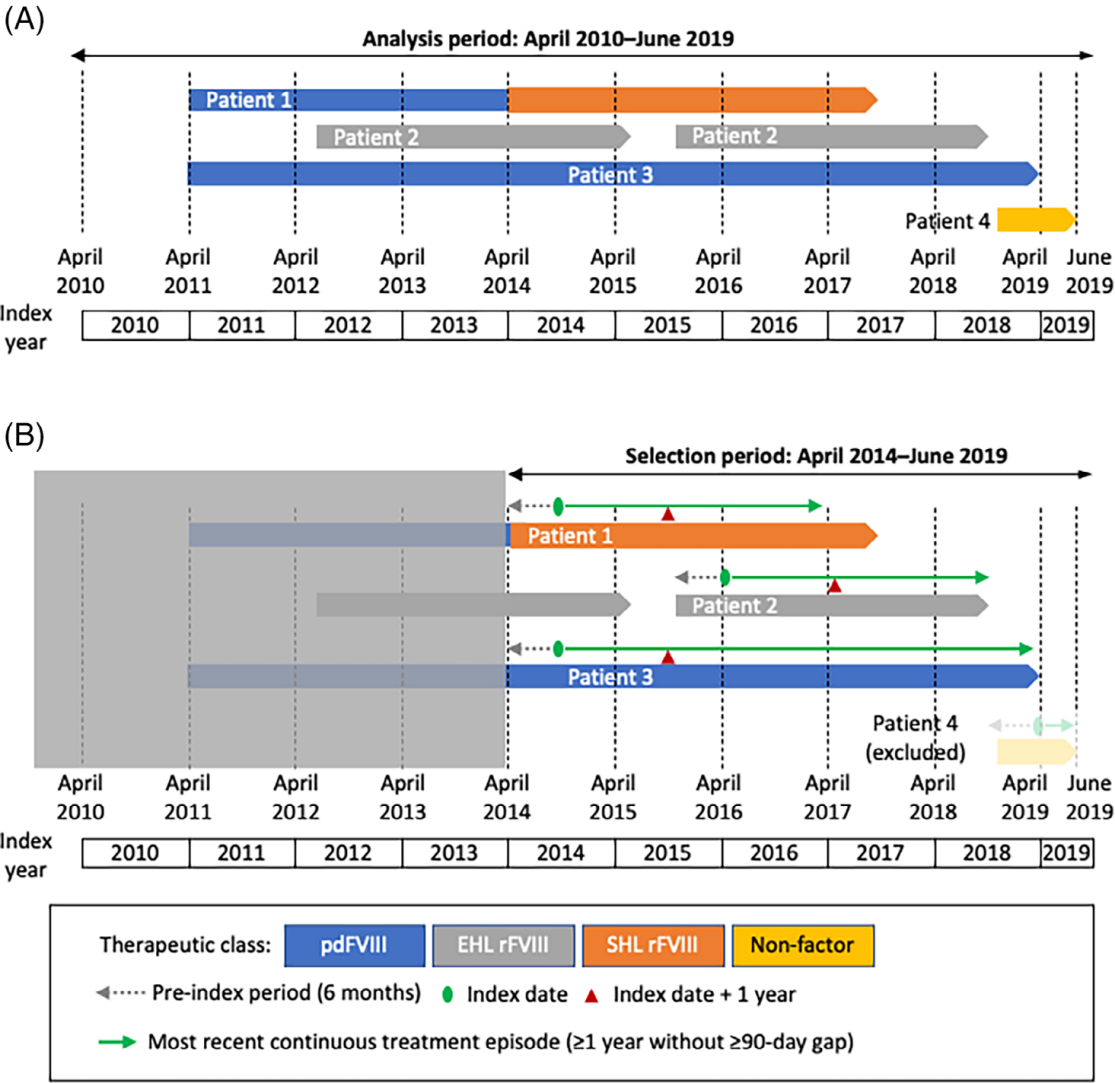
Study design for Objective 1 (A) and Objective 2 (B). (A) Study design for Objective 1. The prescribed medicine in each index year was counted and categorized. Block arrows represent: (Patient 1) a patient switching from pdFVIII to EHL rFVIII; (Patient 2) a patient treated with EHL with a break in continuous care; (Patient 3) a patient treated continuously with pdFVIII; (Patient 4) a patient receiving non‐factor (ie, emicizumab) treatment. (B) Study design for Objective 2. In the selection period 2014–2019, the most recent continuously prescribed hemophilia agent (≥1 year, without 90‐day gap) after index date was longitudinally analyzed for annual cumulative dose and the medical history of the patient in the pre‐index period was collected. Index date was defined as the first recorded treatment in most recent episode. Patient 4 is excluded because the duration of continuous treatment was less than 1 year. EHL, extended half‐life; FVIII, factor VIII; pdFVIII, plasma‐derived FVIII; rFVIII, recombinant FVIII; SHL, standard half‐life

This study was a retrospective, observational database analysis to evaluate the patterns of treatment in Japanese patients with hemophilia A. Data derived from anonymized reimbursements were extracted from the JMDC (formerly known as Japan Medical Data Centre) and Medical Data Vision (MDV) databases between April 1, 2010 and June 30, 2019 (the selection period). Patients were enrolled into two cohorts with distinct study populations to separately explore Objectives 1 (therapeutic agent selection) and 2 (annual cumulative dose; Figure 1A,B, respectively).

The patient cohort for Objective 1 (ie, source cohort) were those with at least one record of receiving hemophilia A treatment based on an International Classification of Diseases, Tenth Revision (ICD‐10) diagnostic code of D66 during the selection period. The index date was the earliest of the first hereditary FVIII deficiency diagnosis or the first recorded claim for prescription of coagulation factors. For Objective 2 (ie, derived cohort), patients from the source cohort with at least 1 year of continuous prescriptions for a hemophilia A treatment without a gap of 90 days or more were selected and the most recent, continuous 1‐year treatment episode was analyzed. The index date was the date of the first recorded hemophilia A treatment prescription in the most recent treatment episode.

### 2.2 Data sources

The JMDC and MDV databases were used to capture patient characteristics and prescription claims data. JMDC is a payer‐based administrative database that maintains inpatient and outpatient medical and pharmacy claims, and enrolment information for salaried workers and their families for the Healthcare Insurance Association (Table S1). For the defined study selection period, the JMDC represented approximately 7.3 million covered lives from several private health insurance plans in Japan. The MDV database maintains standardized healthcare insurance claims data from approximately 27.5 million patients treated in acute‐care hospital settings using the Japanese Diagnosis Procedures Combination (DPC) fixed‐payment reimbursement system. Therefore, the JMDC database mainly represents medical care in small outpatient clinics, whereas the MDV database represents both out‐ and inpatient medical care in large hospitals. For both databases, prescriptions of blood coagulation factors of interest were identified using European Pharmaceutical Market Research Association (EphMRA) and World Health Organization (WHO) Anatomical Therapeutic Chemical (ATC) classification codes as follows: FVIII, EphMRA ATC code B2D1 and WHO ATC codes B02BD02 and B02B06; and emicizumab, EphMRA ATC code B2D1 and WHO ATC code B02BX06. Comorbidities and hematological tests were identified using ICD‐10 diagnostic codes (Tables S2 and S3).

### 2.3 Patients

This study analyzed two distinct cohorts; for Objective 1, the source cohort included male patients with one or more inpatient or outpatient visits for hemophilia A, based on the use of the D66 hereditary FVIII deficiency ICD‐10 code for patients who had a record of the administration of blood coagulation factors of interest from any of the four categories of pdFVIII, SHL rFVIII, EHL rFVIII, or non‐factor (ie, emicizumab) during the analysis period (April 2010 to June 2019, as above). Patients who received an anti‐inhibitor coagulant complex (marketed as Feiba NF intravenous), eptacog alfa, or freeze‐dried activated human plasma‐derived factor VIIa and X (marketed as Biclot) were excluded. The derived cohort for Objective 2 included patients identified in the source cohort who had at least 1 year of continuous prescriptions without a 90‐day gap period of a hemophilia A treatment between April 2014 and June 2019, and who had a continuous enrolment in the database for at least 6 months before the index date.

### 2.4 Outcomes

For Objective 1, the outcomes were the number and proportion of patients taking a hemophilia A treatment by drug category of interest at index date overall, and with additional sub‐cohorts by type of medical facility and database. For Objective 2, the one‐year cumulative dose after 2014—the fiscal year following the introduction of the latest (2013) guideline—was analyzed as a continuous variable with additional sub‐cohorts defined according to the type of medical facility and patient's age. For medical facility type, specialized vs nonspecialized medical facilities were defined as those at which five or more vs fewer than five patients with hemophilia A were prescribed blood coagulation factors of interest within the fiscal year of April 2018 through March 2019, respectively. The threshold of five patients annually was set based on a survey of hemophilia patients in Japan that suggested that non‐specialized clinics (defined as fewer than five patients annually) may provide sub‐optimal treatment.^13^ The possibility that facilities with no specialized physicians may provide care for some patients, and a preliminary analysis of the databases that found a high proportion of patients were treated in clinics with fewer than five patients annually, also informed this decision. For both the JMDC and MDV databases, the number of patients who were prescribed pdFVIII, SHL rFVIII, EHL rFVIII or non‐factor treatment by facility type per fiscal year was recorded.

For Objective 2, patients were evaluated for related comorbidities and hematological tests. Complications specific to hemophilia A were major hemophilia bleeding (major bleeding, joint bleeding, muscular hemorrhage, intracerebral hemorrhage, coagulopathy, purpura, hematoma and other bleeding conditions), liver disease (chronic viral hepatitis, cirrhosis, liver cancer), joint lesions (hemophilia arthropathy and hemophilia arthritis, synovitis), and thrombotic disorders (cerebral infarction, blood deficiency heart disease).

### 2.5 Data analysis

Baseline characteristics were analyzed using descriptive statistics, with quantitative variables described according to mean and standard deviation (SD), and categorical variables summarized according to the number and percentage of patients in each category. For the JMDC database, an algorithm was developed to handle missing data and consisted of approximating the exact date of a claim using the first date of events attached to a claim when available. The full date of a claim was imputed using the first full date among dates of procedures, prescriptions or admissions attached to the claim. No data imputation was planned for the MDV database, and no data imputation was performed for other study variables in either database. Drug categories were calculated inclusively, that is, patients receiving multiple blood coagulation factors of interest were counted several times. For patients in whom all prescriptions of the most recent treatment episode were recorded within the 1‐year period, the annual cumulative dose (Objective 2) was calculated according to the following formula:

$$\text{Annual Cumulative dose}=\text{Dose}\left(\mathrm{Rx}1\right)+\text{Dose}\left(\mathrm{Rx}2\right)+\ldots +\text{Dose}\left(\mathrm{Rxn}\right)$$

For patients with prescriptions outside this period (ie, earlier prescriptions), the cumulative dose was adjusted according to prescription duration inside and outside the 1‐year period, according to the following formula:

$$\text{Cumulative dose}=\text{Dose}\left(\mathrm{Rx}1\right)\;x\frac{\left(\mathrm{Rx}1\;\mathrm{end}–\mathrm{one}\;\text{year period start}\right)}{\mathrm{Rx}1\;\mathrm{end}–\mathrm{Rx}1\;\text{start}}+\text{Dose}\left(\mathrm{Rx}2\right)+\ldots \text{Dose}\left(\mathrm{Rxn}\right)$$

The mean dose per treatment was calculated as the mean annual cumulative dose divided by the product of 52 weeks multiplied by the frequency of administration. We set the precondition as for the frequency of administration three times weekly for pdFVIII and SHL rFVIII, and twice weekly for EHL rFVIII here based on general information in the package inserts. Data management was performed using SAS software, version 9.3.

### 2.6 Ethical considerations

This study was based on anonymized secondary use data; an ethical review was not considered necessary according to the local ethical guidelines, and such a review was not performed.

## 3 RESULTS

### 3.1 Patient disposition

**FIGURE 2:**
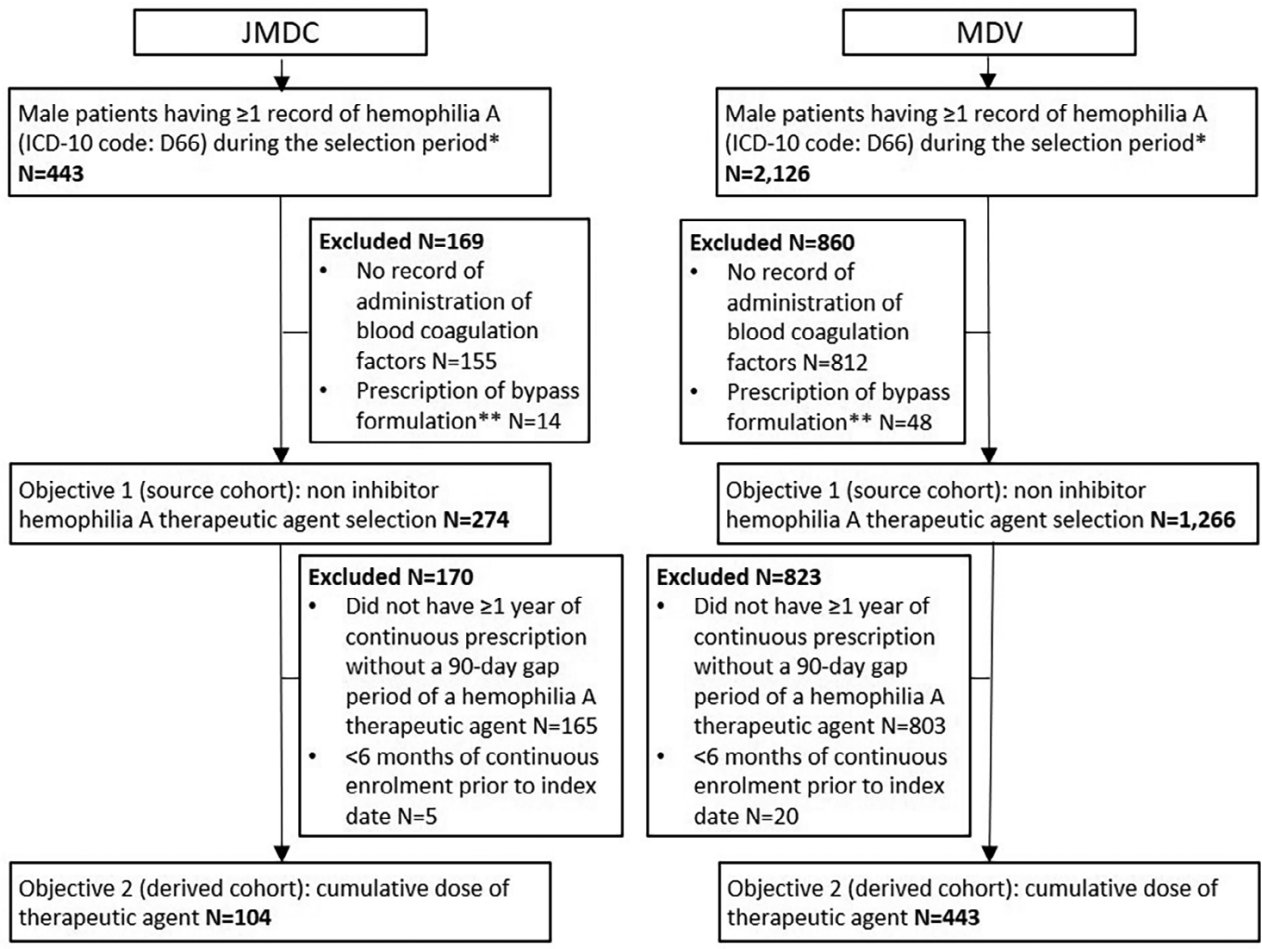
Patient disposition for the JMDC and MDV databases. *April 1, 2010 to June 30, 2019; **aPCC, rFVIIa, pdFVIIa/FX. aPCC, activated prothrombin complex concentrate; FX, factor X; HA, hemophilia A; ICD‐10, International Classification of Diseases, Tenth Revision; MDV, Medical Data Vision; pdFVIIa, plasma‐derived factor VIIa; rFVIIa, recombinant factor VIIa

Patient disposition is summarized in Figure 2. Briefly, 443 and 2126 male patients were identified from the JMDC and MDV databases, respectively, of which 169 and 860 patients, respectively, were excluded predominantly due to no record of the administration of a blood coagulation factor of interest; 274 and 1266 patients, respectively, met all inclusion criteria for Objective 1. Of these, 170 of 274 patients (62% of Objective 1) and 823 of 1266 patients (63% of Objective 1), respectively, were excluded from the analyses for Objective 2. The majority of these patients were excluded because they did not have at least 1 year of continuous (without a 90‐day gap) prescription of a hemophilia A treatment. Consequently, 104 and 443 patients, respectively, met all inclusion criteria for Objective 2. The clinical characteristics of the patients meeting the criteria for Objective 2 are summarized in Table 1 (JDMC) and Table 2 (MDV). The mean age was 22.9 years in the JDMC population and 31.5 years in the MDV population. In both populations, most patients (75.0% and 57.3% in JMDC and MDV, respectively) were younger than 35 years.

**TABLE 1:** Patient demographic and clinical characteristics at index‐date (JMDC, Objective 2)

Characteristics	All	Medical facility category		Age			
		Nonspecialized (<5 patients)	Specialized (≥5 patients)	<12	12–35	36–60	>60
	N = 104	N = 99	N = 5	N = 35	N = 43	N = 24	N = 2
Age, in years							
Age at index date, mean (SD)	22.9 (16.1)	23.4 (16.1)	15.4 (16.3)	NA	NA	NA	NA
Age at index date by categories, n (%)							
<12	35 (33.7%)	32 (32.3%)	3 (60.0%)	NA	NA	NA	NA
12–35	43 (41.3%)	42 (42.4%)	1 (20.0%)	NA	NA	NA	NA
36–60	24 (23.1%)	23 (23.2%)	1 (20.0%)	NA	NA	NA	NA
>60	2 (1.9%)	2 (2.0%)	0 (0.0%)	NA	NA	NA	NA
Comorbidities and HA‐specific complications, n (%)							
Hemophilia bleeding	17 (16.3%)	15 (15.2%)	2 (40.0%)	10 (28.6%)	6 (14.0%)	1 (4.2%)	0 (0.0%)
Liver disease	30 (28.8%)	29 (29.3%)	1 (20.0%)	1 (2.9%)	4 (9.3%)	23 (95.8%)	2 (100%)
Joint lesions	2 (1.9%)	2 (2.0%)	0 (0.0%)	0 (0.0%)	1 (2.3%)	1 (4.2%)	0 (0.0%)
Thrombotic disorder	2 (1.9%)	1 (1.0%)	1 (20.0%)	2 (5.7%)	0 (0.0%)	0 (0.0%)	0 (0.0%)
Diabetes	4 (3.8%)	4 (4.0%)	0 (0.0%)	0 (0.0%)	0 (0.0%)	3 (12.5%)	1 (50.0%)
Hypertension	10 (9.6%)	9 (9.1%)	1 (20.0%)	0 (0.0%)	1 (2.4%)	7 (28%)	2 (100%)
Hyperlipidemia	7 (6.7%)	7 (7.1%)	0 (0.0%)	0 (0.0%)	1 (2.3%)	6 (25.0%)	0 (0.0%)
Chronic kidney failure	2 (1.9%)	2 (2.0%)	0 (0.0%)	0 (0.0%)	1 (2.3%)	1 (4.2%)	0 (0.0%)
Diagnostic testing associated with HA, n (%)							
Hematological test	65 (62.5%)	62 (62.6%)	3 (60.0%)	25 (71.4%)	26 (60.5%)	13 (54.2%)	1 (50.0%)
Hemophilia A drug classes, n (%)							
A: pdFVIII	4 (3.8%)	4 (4.0%)	0 (0.0%)	0 (0.0%)	3 (7.0%)	1 (4.2%)	0 (0.0%)
B: SHL rFVIII	63 (60.6%)	62 (62.6%)	1 (20.0%)	25 (71.4%)	24 (55.8%)	12 (50.0%)	2 (100%)
C: EHL rFVIII	37 (35.6%)	33 (33.3%)	4 (80.0%)	10 (28.6%)	16 (37.2%)	11 (45.8%)	0 (0.0%)
D: Non‐factor	0 (0.0%)	0 (0.0%)	0 (0.0%)	0 (0.0%)	0 (0.0%)	0 (0.0%)	0 (0.0%)
Medical facility type							
Nonspecialized (<5 patients)	99 (95.2%)	NA	NA	32 (91.4%)	42 (97.7%)	23 (95.8%)	2 (100%)
Specialized (≥5 patients)	5 (4.8%)	NA	NA	3 (8.6%)	1 (2.3%)	1 (4.2%)	0 (0.0%)

*Note*: Percentages are calculated based on total population (N) for each column.

Abbreviations: EHL, extended half‐life; FVIII, factor VIII; HA, hemophilia A; MRI magnetic resonance imaging; NA, not applicable; pd FVIII, plasma‐derived FVIII; rFVIII, recombinant FVIII; SD, standard deviation; SHL, standard half‐life.

**TABLE 2:** Patient demographic and clinical characteristics at index‐date (MDV, Objective 2)

Characteristics	All	Medical facility category		Age			
		Nonspecialized (<5 patients)	Specialized (≥5 patients)	<12	12–35	36–60	>60
	N = 443	N = 73	N = 370	N = 96	N = 158	N = 136	N = 53
Age, in years							
Age at index date, mean (SD)	31.5 (20.8)	30.6 (21.1)	31.7 (20.7)	NA	NA	NA	NA
Age at index date by categories, n (%)							
<12	96 (21.7%)	15 (20.5%)	81 (21.9%)	NA	NA	NA	NA
12–35	158 (35.7%)	32 (43.8%)	126 (34.1%)	NA	NA	NA	NA
36–60	136 (30.7%)	15 (20.5%)	121 (32.7%)	NA	NA	NA	NA
>60	53 (12.0%)	11 (15.1%)	42 (11.4%)	NA	NA	NA	NA
Comorbidities and HA‐specific complications, n (%)							
Hemophilia bleeding	76 (17.2%)	13 (17.8%)	63 (17.0%)	22 (22.9%)	26 (16.5%)	14 (10.3%)	14 (26.4%)
Liver disease	189 (42.7%)	17 (23.3%)	172 (46.5%)	1 (1.0%)	22 (13.9%)	118 (86.8%)	48 (90.6%)
Joint lesions	3 (0.7%)	0 (0.0%)	3 (0.8%)	0 (0.0%)	0 (0.0%)	3 (2.2%)	0 (0.0%)
Thrombotic disorder	16 (3.6%)	0 (0.0%)	16 (4.3%)	0 (0.0%)	3 (1.9%)	9 (6.6%)	4 (7.5%)
Diabetes	59 (13.3%)	9 (12.3%)	50 (13.5%)	0 (0.0%)	6 (3.8%)	31 (22.8%)	22 (41.5%)
Hypertension	85 (19.2%)	13 (17.8%)	72 (19.5%)	0 (0.0%)	2 (1.3%)	44 (32.4%)	39 (73.6%)
Hyperlipidemia	51 (11.5%)	4 (5.5%)	47 (12.7%)	1 (1.0%)	5 (3.2%)	30 (22.1%)	15 (28.3%)
Chronic kidney failure	11 (2.5%)	2 (2.7%)	9 (2.4%)	0 (0.0%)	1 (0.6%)	7 (5.1%)	3 (5.7%)
Diagnostic testing associated with HA, n (%)							
Hematological test	321 (72.5%)	41 (56.2%)	280 (75.7%)	62 (64.6%)	104 (65.8%)	107 (78.7%)	48 (90.6%)
Hemophilia A drug classes, n (%)							
A: pdFVIII	33 (7.4%)	5 (6.8%)	28 (7.6%)	0 (0.0%)	9 (5.7%)	16 (11.8%)	8 (15.1%)
B: SHL rFVIII	274 (61.9%)	45 (61.6%)	229 (61.9%)	76 (79.2%)	98 (62.0%)	70 (51.5%)	30 (56.6%)
C: EHL rFVIII	135 (30.5%)	22 (30.1%)	113 (30.5%)	20 (20.8%)	51 (32.3%)	49 (36.0%)	15 (28.3%)
D: Non‐factor	1 (0.2%)	1 (1.4%)	0 (0.0%)	0 (0.0%)	0 (0.0%)	1 (0.7%)	0 (0.0%)
Medical facility type							
Nonspecialized (<5 patients)	73 (16.5%)	NA	NA	15 (15.6%)	32 (20.3%)	15 (11.0%)	11 (20.8%)
Specialized (≥5 patients)	370 (83.5%)	NA	NA	81 (84.4%)	126 (79.7%)	121 (89.0%)	42 (79.2%)

*Note*: Percentages are calculated based on total population (N) for each column.

Abbreviations: EHL, extended half‐life; FVIII, factor VIII; HA, hemophilia A; MRI, magnetic resonance imaging; NA, not applicable; pd FVIII, plasma‐derived FVIII; rFVIII, recombinant FVIII; SD, standard deviation; SHL standard half‐life.

### 3.2 Objective 1: Therapeutic selection

**FIGURE 3:**
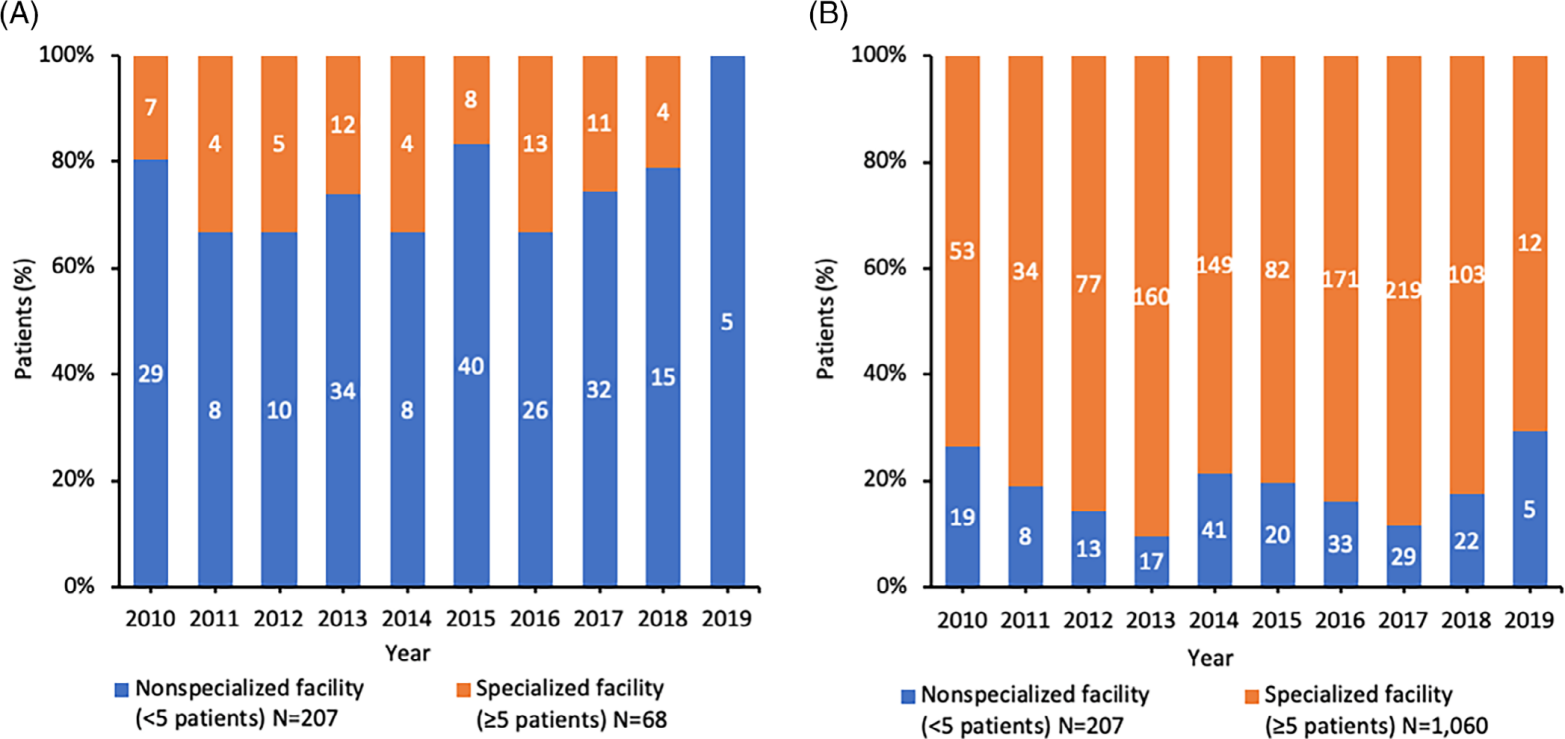
Percentage of patients meeting the inclusion criteria of Objective 1 (therapy comparison) in the JMDC (A) and MDV (B) databases for fiscal years 2010–2019. Labels are absolute patient numbers. Total patient number in JMDC includes one patient who was counted twice due to having two different hemophilia A treatments at index date

In the JMDC database, 68 of 275 (24.8%) patients were treated in specialized medical facilities in the period evaluated (Figure 3A). The absolute number of patients receiving specialized care in the JMDC population was small for all years assessed. For example, in the April 2018 to March 2019 fiscal year, 19 patients in total received treatment with a coagulation factor of interest, four of whom received treatment in a specialized medical facility. Due to these small patient numbers, meaningful comparisons for the two study objectives could not be performed using the JMDC population.

**FIGURE 4:**
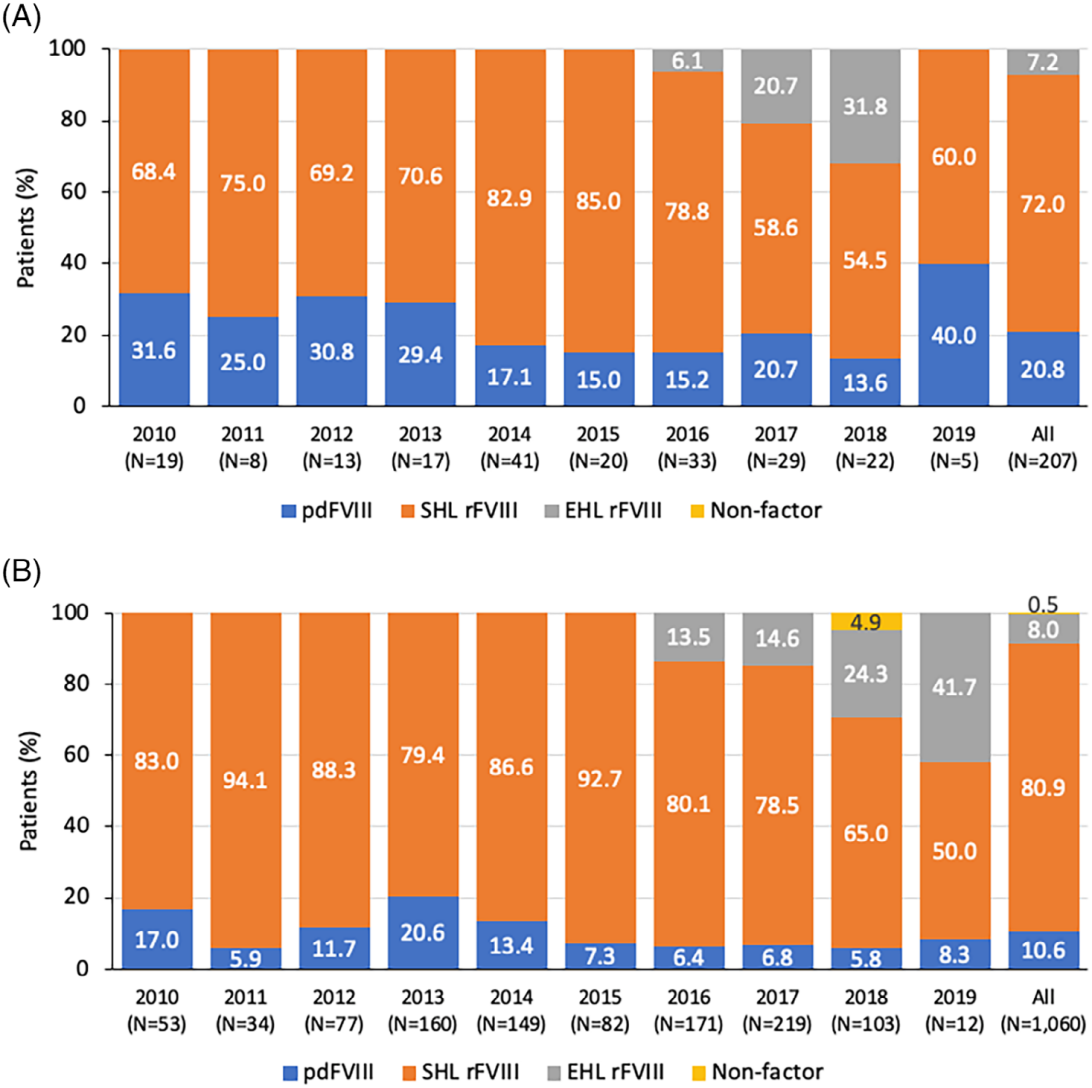
Types of anti‐hemophilia A treatments in patients in the MDV database treated in (A) nonspecialized and (B) specialized medical facilities. EHL, extended half‐life; FVIII, factor VIII; pdFVIII, plasma‐derived FVIII; rFVIII, recombinant FVIII; SHL, standard half‐life

The MDV database yielded a larger number of patients meeting the inclusion criteria for all years assessed, with 207 (16.3%) and 1060 (83.7%) patients receiving care in nonspecialized and specialized medical facilities, respectively (Figure 3B). Of the 22 patients treated in a nonspecialized medical facility in 2018, three (13.6%) received pdFVIII compared with six of 103 (5.8%) treated in a specialized medical facility (Figure 4A,B). During the entire period evaluated, a higher percentage of patients in nonspecialized medical facilities (20.8%) received pdFVIII when compared with those at specialized medical facilities (10.6%). The predominant form of FVIII replacement was SHL rFVIII in both settings, used by 54.5% and 65.0% of patients in nonspecialized and specialized settings, respectively in 2018; use of EHL rFVIII increased from 6.1% (nonspecialized) and 13.5% (specialized) in 2016 to 31.8% and 24.3% in 2018, respectively. The use of non‐factor treatment was rare and reported only in a small number of patients in specialized medical facilities.

### 3.3 Objective 2: Annual cumulative dose

**FIGURE 5:**
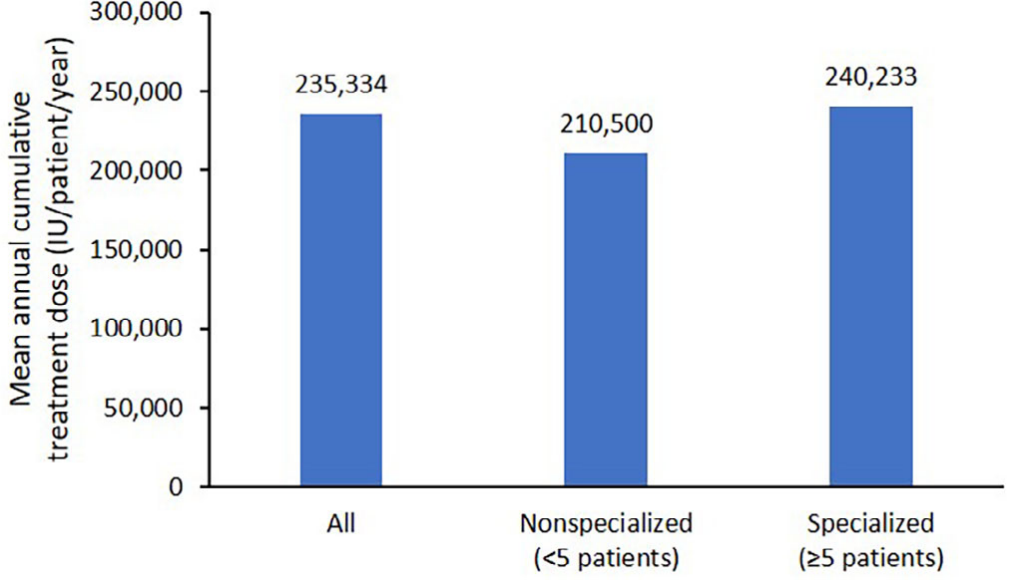
Mean annual cumulative dose of all blood coagulation factors of interest in the MDV database by facility type. IU, international units

Compared with nonspecialized medical facilities, patients in the MDV database who were treated at specialized medical facilities with any blood coagulation factor received a 14% higher mean annual cumulative dose (Figure 5). In the MDV database, patients with the most recent continuous 1‐year treatment were prescribed a mean annual cumulative treatment dose of 235,334 international units (IU) per patient per year. Mean annual cumulative doses of pdFVIII, SHL FVIII and EHL FVIII were numerically higher in specialized vs nonspecialized medical facilities (Table 3). In the MDV data, the similar age distribution was observed in both specialized medical facilities and nonspecialized medical facilities (Figure S1), suggesting the age distribution between specialized and non‐specialized facilities was almost the same, and therefore unlikely to explain differences between the facility types (bodyweight data were not available in the databases). Mean doses per treatment were 1103 IU (nonspecialized) and 1275 IU (specialized) for pdFVIII, 1081 IU (nonspecialized) and 1313 IU (specialized) for SHL rFVIII, and 3020 IU (nonspecialized) and 3097 IU (specialized), for EHL rFVIII.

**TABLE 3:** Annual mean cumulative dose of hemophilia A therapies (MDV database)

Medical facility type	Nonspecialized (<5 patients)		Specialized (≥5 patients)	
Therapy class	n	Mean annual cumulative dose, IU (SD)	n	Mean annual cumulative dose, IU (SD)
pdFVIII	5	172,000 (188,760)	28	198,911 (138,295)
SHL rFVIII	45	168,672 (93,390)	229	204,873 (152,880)
EHL rFVIII	22	314,114 (147,104)	113	322,132 (191,296)

Abbreviations: EHL, extended half‐life; FVIII, factor VIII; IU, international units; pdFVIII, plasma‐derived FVIII; rFVIII, recombinant FVIII; SD, standard deviation; SHL, standard half‐life.

### 3.4 Other findings

Data from the derived cohorts were used to evaluate rates of medical procedures and comorbidities. Among JMDC patients, 62 of 99 (62.6%) patients received hematological testing at nonspecialized medical facilities as did three of five patients at specialized medical facilities (Table 1). In the MDV database, fewer patients at nonspecialized medical facilities received hematological tests than at specialized medical facilities (41 of 73 [56.2%] and 280 of 370 [75.7%] patients, respectively; Table 2). The proportion of patients with comorbidities was similar between specialized and nonspecialized medical facilities for most comorbidity types (Tables 1 and 2), except for liver disease and hyperlipidemia, which were higher among patients in specialized medical facilities.

## 4 DISCUSSION

The findings of this claims‐based retrospective cohort study suggest that there are opportunities to improve the management of patients with hemophilia A in Japan. Although the “Hemophilia Center” concept was proposed by the Japanese Society on Thrombosis and Hemostasis in 2010, the findings of our study suggest that three out of every four patients received care in nonspecialized medical facilities in the JMDC population. In the MDV cohort, the majority of patients were treated in specialized medical facilities across the period evaluated, but the MDV database is unlikely to be representative of the overall healthcare system as it only includes larger hospitals participating in the DPC reimbursement system, and does not include smaller hospitals and clinics. We are unaware of any factors that would motivate a patient preference for treatment in smaller facilities; we speculate that access to specialized facilities, which tend to be bigger hospitals in the center of cities, may be difficult for patients who reside elsewhere in Japan.

While the number of patients included in the JMDC population was too low to allow a meaningful comparison of FVIII therapeutic choices by facility type, analysis of the MDV population suggested that only a small minority of patients were treated with pdFVIII, including 20.8% of patients in specialized medical facilities and 10.6% of patients in nonspecialized medical facilities. The predominant form of treatment was SHL rFVIII in both settings, and the use of EHL rFVIII increased steadily in both settings from 2016 onwards. A plausible explanation for the lower use of pdFVIII and higher use of rFVIII and non‐FVIII therapies in specialized medical facilities could be a greater awareness and familiarity with these novel options among physicians with a higher case load.

### 4.1 Annual doses of FVIII replacement therapies

Patients in the MDV database received a numerically higher mean annual cumulative dose of pdFVIII or SHL rFVIII when treated at specialized medical facilities compared with nonspecialized medical facilities, whereas doses of EHL rFVIII were smaller. The comparison of patient ages between those treated in specialized and nonspecialized settings suggests that differences in patient body weight (ie, a higher proportion of younger, smaller patients in nonspecialized medical facilities) do not explain this difference. The higher annual cumulative dose of EHL rFVIII compared with pdFVIII or SHL rFVIII observed in both settings is consistent with the higher dose recommended in the product information of EHL rFVIII products. The product information provides essential reference information to the physician, and compared with pdFVIII and SHL rFVIII, the package inserts of the newer EHL rFVIII products specify a stricter fixed prophylaxis regimen. These data suggest that physicians at nonspecialized facilities are following the dosing recommendations in the product information of EHL rFVIII products, resulting in similar annual cumulative doses to patients treated at specialized facilities.

### 4.2 Frequency of hematological testing

Among the patients receiving continuous prescriptions in a healthcare setting, the MDV database indicated that annual hematological testing was conducted in fewer than 60% of patients who were treated at nonspecialized medical facilities, compared with 75% of patients at specialized medical facilities. The absence of hematological testing in many patients raises concern as testing is critical, not only for screening and diagnosis of hemophilia A, but also for monitoring response to treatment.^14^, ^15^ The 2013 Japanese Society on Thrombosis and Hemostasis guidelines recommend patients undergo hematological testing to ensure plasma FVIII levels meet the required thresholds, set appropriate dosing and frequency of prophylactic treatment, and detect the presence of inhibitors.^16^ These deficiencies could be addressed by emphasizing the use of the current Japanese guidelines that promote the provision of care in specialized medical facilities and highlight the importance of regular testing. Awareness and educational programs aimed at physicians in nonspecialized medical facilities who treat hemophilia A patients can also contribute to this goal.

### 4.3 Continuity of hemophilia care

In evaluating patient disposition, the large proportion of hemophilia A patients excluded from Objective 1—170/274 (62%) in the JMDC population and 803/1266 (63%) in the MDV population—indicates that the majority of hemophilia A patients are not receiving continuous prescriptions of FVIII replacement as defined in our study. This proportion is higher than the 35% reported in the Nationwide Survey on Coagulation Disorders 2018.^3^ However, this comparison must be regarded with caution as the definition of continuous prescription used in our study—at least 1 year of use with no gap of 90 days or longer—is likely to differ from the survey definition of “regular hospital care.” Our finding that there is a high proportion of patients not receiving continuous prescriptions in both databases suggests there are potential opportunities to improve care for patients with hemophilia A in Japan. More importantly, because continuous prophylaxis supported by continuous prescription of factor products is a recommended approach for prevention of hemophilic arthropathy in patients with hemophilia A, our result raises concerns that many Japanese hemophilia A patients receive insufficient prophylaxis. Moreover, prophylactic regimens, notably the target FVIII level, cannot be generalized because of individual differences in FVIII clearance and joint disease activity among patients. Therefore, regular optimized care, including hematological testing and individualized prophylaxis, may be the desired approach.

### 4.4 Strengths and limitations

The strengths of this study are the use of databases with large sample sizes and continuous long‐term patient data. Limitations include the retrospective, observational design which may result in selection bias, and the inclusion criteria may not be representative of all patients with hemophilia A in Japan. Although the two databases used both have large populations, they may not cover all hemophilia A patients in Japan. The inclusion criteria are based on prescription, rather than administration, and the study did not factor in medical history such as differences in levels of bleeding control at baseline. Information on the severity of bleeding experienced by patients was not available in the databases used. Another limitation is the arbitrary distinction between specialized and nonspecialized medical facilities based on the number of patients treated. The study design does not permit the analysis of differences between specialized and nonspecialized for statistical significance. We did not exclude patients undergoing surgery from the analysis for Objective 2, however, the effect such patients might have would be limited because we calculated consumption as a one‐year average. Furthermore, patients who need surgery may be more likely to attend specialized centers, which may contribute to the greater consumption of FVIII replacements seen in patients attending specialized facilities. The definition of continuous prescription as at least 1 year of continuous treatment without a gap of 90 days or longer also removes some patients with infrequent prophylaxis from our population.

There are differences in database characteristics. JMDC captures nationwide health insurance claims data from health insurance providers. This includes claims of outpatients, inpatients, DPC reimbursements and dispensing. The data include employees and their families in relatively large corporations in urban areas but contains little data for patients older than 65 years and no data for those older than 75 years. Individual data can be linked if patients transfer to other facilities, but patients cannot be traced if they change their insurer. The MDV database captures claims data of inpatients and outpatients from DPC hospitals which are relatively large hospitals providing acute medical care, and this database includes patients of all ages. Among the MDV population, data from multiple hospitals cannot be combined, which may lead to duplication of data. This study is the first attempt to assess the management of hemophilia A in different clinical settings in Japan by retrospective use of claims databases. However, for more accurate information, it would be desirable to establish a nationwide patient registry for individuals receiving hemophilia A treatment, as recommended in a 2018 publication by The Asia‐Pacific Haemophilia Working Group.^17^

## 5 CONCLUSIONS

Our data suggest that many Japanese patients with hemophilia A may receive suboptimal care, with a high proportion of patients not partaking in continuous prophylaxis, and a lack of hematological testing among those who do receive care. Patients are treated predominantly with rFVIII products, but many are treated with pdFVIII, particularly in a nonspecialized setting. We suggest emphasizing the current local guidelines and educational programs, especially in nonspecialized facilities, that highlight the importance of directing patients to receive care in specialized centers. Although this study demonstrates the limitations of using a claims database to monitor hemophilia care, it also shows that such an approach can yield insights into nationwide hemophilia care that would otherwise be unavailable.

## CONFLICT OF INTEREST

Ei Kinai has no conflicts of interest to declare. Midori Ono, Akinori Oh, Mihoko Ota, Yasuo Myaguchi, and Hitoshi Ueda are employees of Takeda Pharmaceutical Company Limited. Midori Ono, Akinori Oh, Mihoko Ota, and Hitoshi Ueda own shares in Takeda Pharmaceutical Company Limited.

## AUTHOR CONTRIBUTIONS

Conceptualization: Ei Kinai, Midori Ono, Hitoshi Ueda, Akinori Oh

Data Curation: Ei Kinai, Midori Ono, Hitoshi Ueda, Mihoko Ota; Akinori Oh

Formal Analysis: Mihoko Ota, Akinori Oh

Investigation: Ei Kinai; Midori Ono; Hitoshi Ueda; Akinori Oh

Methodology: Ei Kinai; Midori Ono; Hitoshi Ueda; Mihoko Ota; Akinori Oh

Project Administration: Midori Ono, Yasuo Miyaguchi

Resources: Mihoko Ota

Supervision: Ei Kinai, Hitoshi Ueda; Mihoko Ota

Validation: Ei Kinai

Writing‐Original Draft: Midori Ono, Hitoshi Ueda

Writing‐Review & Editing: Ei Kinai, Midori Ono, Hitoshi Ueda, Mihoko Ota, Akinori Oh, Yasuo Miyaguchi

All authors have read and approved the final version of the manuscript.

## TRANSPARENCY STATEMENT

Midori Ono confirms that this manuscript is an honest, accurate, and transparent account of the study being reported; that no important aspects of the study have been omitted; and that any discrepancies from the study as planned have been explained.

## Supporting information


**Table S1.** Description of JMDC and MDV databases

**Table S2.** Diagnostic procedure codes

**Table S3.** Complication and comorbidity codes

**Figure S1.** Patients' age distribution in specialized and nonspecialized medical facilities (MDV database).

## Data Availability

Data used in this study are available from JMDC and MDV but were used under license for the current study; therefore, restrictions apply, and the data are not publicly available. For inquiries about access to the data set used in this study, please contact JMDC (https://www.jmdc.co.jp) or MDV (https://www.mdv.co.jp/mdv_database/english/).
