# Supplementary material for: Analysis of nationwide hemophilia care: A cohort study using two Japanese healthcare claims databases
Source: Health Sci Rep. 2022 Jan 27;5(1):e498. doi: 10.1002/hsr2.498 (PMC8795212; doi:10.1002/hsr2.498)
Supplement: Supplementary file 1 — Table S1. Description of JMDC and MDV databases [file HSR2-5-e498-s004.pdf]

Supplementary Table 1. Description of JMDC and MDV databases

	JMDC	MDV
**Data source**	Payer-Based database （Healthcare Insurance associations)	Diagnostic Procedures Combination (DPC) hospital database
**Sample sizes (June 2019)**	Approximately 7,300,000 people (accumulative) from more than 200 operators	27,570,000 people from 383 hospitals
**Data contents**	• Inpatient and outpatient claims<br>• Health check-up results<br>• Enrolment information from health insurance associations	• Inpatient and outpatient claims<br>• Health check-up results from DPC hospitals

DPC, diagnosis procedures combination; JMDC, Japan Medical Data Center; MDV, Medical Data Vision.
