# Supplementary material for: Analysis of nationwide hemophilia care: A cohort study using two Japanese healthcare claims databases
Source: Health Sci Rep. 2022 Jan 27;5(1):e498. doi: 10.1002/hsr2.498 (PMC8795212; doi:10.1002/hsr2.498)
Supplement: Supplementary file 2 — Table S2. Diagnostic procedure codes [file HSR2-5-e498-s002.pdf]

Supplementary Table 2. Diagnostic procedure codes

Procedure category	JMDC claims database		MDV database	
	Standardized procedure code	Procedure name	Standardized procedure code	Procedure name
Hematological test	160182410	Coagulation factor inhibitor, qualitative	160182410	Coagulation factor inhibitor, qualitative
Hematological test	160015410	Coagulation factor inhibitor, FVIII	160015410	Coagulation factor inhibitor, FVIII
Hematological test	160016210	Coagulation FVIII	160016210	Coagulation FVIII
Hematological test	160012310	Activated partial thromboplastin time	160012310	Activated partial thromboplastin time

FVIII, factor VIII; MDV, Medical Data Vision.
