# Supplementary material for: Analysis of nationwide hemophilia care: A cohort study using two Japanese healthcare claims databases
Source: Health Sci Rep. 2022 Jan 27;5(1):e498. doi: 10.1002/hsr2.498 (PMC8795212; doi:10.1002/hsr2.498)
Supplement: Supplementary file 3 — Table S3. Complication and comorbidity codes [file HSR2-5-e498-s001.pdf]

Supplementary Table 3. Complication and comorbidity codes

Complication or comorbidity	Injury/disease name	ICD-10 code (JMDC)	ICD-10 Code (MDV)
Hemophilia bleeding	Ischemic infarction of muscle	M622	M6226
Hemophilia bleeding	Atraumatic compartment syndrome		M6229
Hemophilia bleeding	Traumatic ischemia of muscle	T796	T796
Hemophilia bleeding	Subarachnoid hemorrhage	I60	I601
Hemophilia bleeding	Subdiaphragmatic hemorrhage		I609
Hemophilia bleeding	Intracerebral hemorrhage	I61	I610
Hemophilia bleeding			I611
Hemophilia bleeding			I613
Hemophilia bleeding			I614
Hemophilia bleeding			I615
Hemophilia bleeding			I619
Hemophilia bleeding	Other nontraumatic intracranial hemorrhage	I62	I620
Hemophilia bleeding			I621
Hemophilia bleeding			I629
Hemophilia bleeding	Superficial injury of unspecified body region	T140	T140
Hemophilia bleeding	Open wound of unspecified body region	T141	T141
Hemophilia bleeding	Fracture of unspecified body region	T142	T1420
Hemophilia bleeding	Dislocation, sprain and strain of unspecified body region	T143	T143
Hemophilia bleeding			T144
Hemophilia bleeding			T145
Hemophilia bleeding	Injury of muscles and tendons of unspecified body region	T146	T146
Hemophilia bleeding	Crushing injury and traumatic amputation of unspecified body region	T147	T147
Hemophilia bleeding			T148
Hemophilia bleeding	Injury, unspecified	T149	T149
Hemophilia bleeding	Concussion	S060	S060
Hemophilia bleeding	Diffuse brain injury	S062	S062
Hemophilia bleeding	Closed brain contusion		S0620
Hemophilia bleeding	Focal brain injury	S063	S063
Hemophilia bleeding	Closed local brain contusion		S0630
Hemophilia bleeding	Epidural hemorrhage	S064	S064
Hemophilia bleeding	Closed acute epidural hematoma		S0640
Hemophilia bleeding	Traumatic subdural hemorrhage	S065	S065
Hemophilia bleeding	Closed acute subdural hematoma		S0650
Hemophilia bleeding	Traumatic subarachnoid hemorrhage	S066	S066
Hemophilia bleeding	Obstructive traumatic subarachnoid hemorrhage		S0660
Hemophilia bleeding	Other intracranial injuries	S068	S068

Hemophilia bleeding	Obstructive traumatic intraventricular hemorrhage		S0680
Hemophilia bleeding	Intracranial injury, unspecified	S069	S069
Hemophilia bleeding	Contusion of knee	S800	S800
Hemophilia bleeding	Contusion of other and unspecified parts of lower leg	S801	S801
Hemophilia bleeding	Other superficial injuries of lower leg	S808	S808
Hemophilia bleeding	Contusion of elbow	S500	S500
Hemophilia bleeding	Contusion of other and unspecified parts of forearm	S501	S501
Hemophilia bleeding	Other superficial injuries of forearm	S508	
Liver disease	Chronic viral hepatitis B without delta-agent	B181	B181
Liver disease	Chronic viral hepatitis C	B182	B182
Liver disease	Chronic viral hepatitis, unspecified	B189	B189
Liver disease	Primary biliary cirrhosis	K743	K743
Liver disease	Other and unspecified cirrhosis of liver	K746	K746
Liver disease	Malignant neoplasm of liver and intrahepatic bile ducts / Liver cell carcinoma	C220	C220
Liver disease	Malignant neoplasm of liver and intrahepatic bile ducts / Intrahepatic bile duct carcinoma	C221	C221
Liver disease	Liver cystadenocarcinoma		C227
Liver disease	Malignant neoplasm of liver and intrahepatic bile ducts / Liver, unspecified	C229	C229
Joint lesions	Other synovitis and tenosynovitis	M658	M6589
Joint lesions	Synovitis and tenosynovitis, unspecified	M659	M6594
Joint lesions	Knee synovitis		M6596
Joint lesions	Synovitis		M6599
Thrombotic disorder	Cerebral infarction due to thrombosis of cerebral arteries	I633	I633
Thrombotic disorder	Cardiogenic cerebral embolism		I634
Thrombotic disorder	Cerebral infarction due to unspecified occlusion or stenosis of cerebral arteries	I635	I635
Thrombotic disorder	Other cerebral infarction	I638	I638
Thrombotic disorder	Cerebral infarction, unspecified	I639	I639
Thrombotic disorder	Unstable angina	I200	I200
Thrombotic disorder	Coronary spastic angina		I201
Thrombotic disorder	Other forms of angina pectoris	I208	I208
Thrombotic disorder	Angina pectoris, unspecified	I209	I209
Thrombotic disorder	Acute anterior myocardial infarction		I210
Thrombotic disorder	Acute inferior myocardial infarction		I211
Thrombotic disorder	Acute myocardial infarction		I212
Thrombotic disorder	Myocardial infarction		I214

Thrombotic disorder	Acute myocardial infarction, unspecified	I219	I219
Thrombotic disorder	Subsequent myocardial infarction of unspecified site	I229	
Thrombotic disorder	Other forms of acute ischemic heart disease	I248	I248
Thrombotic disorder	Acute coronary syndrome		I249
Thrombotic disorder	Coronary stenosis		I251
Thrombotic disorder	Old myocardial infarction	I252	I252
Thrombotic disorder	Ischemic cardiomyopathy	I255	I255
Thrombotic disorder	Asymptomatic myocardial ischemia		I256
Thrombotic disorder	Chronic ischemic heart disease, unspecified	I259	I259
Thrombotic disorder	Lower limb thrombophlebitis		I800
Thrombotic disorder	Phlebitis and thrombophlebitis of other deep vessels of lower extremities	I802	I802
Thrombotic disorder	Lower leg phlebitis		I803
Thrombotic disorder	Foot thrombophlebitis		I808
Thrombotic disorder	Phlebitis and thrombophlebitis of unspecified site	I809	I809
Diabetes	Type 2 diabetes mellitus	E11-	E11
Diabetes			E111
Diabetes	Type 2 diabetes mellitus / With renal complications	E112	E112
Diabetes	Type 2 diabetes mellitus / With ophthalmic complications	E113	E113
Diabetes	Type 2 diabetes mellitus / With neurological complications	E114	E114
Diabetes	Type 2 diabetic arteriosclerosis		E115
Diabetes	Type 2 diabetes and joint		E116
Diabetes	Type 2 diabetes mellitus / With multiple complications	E117	E117
Diabetes	Type 2 diabetes mellitus / Without complications	E119	E119
Diabetes	Unspecified diabetes mellitus	E14-	E14
Diabetes			E141
Diabetes	Unspecified diabetes mellitus / With renal complications	E142	E142
Diabetes	Unspecified diabetes mellitus / With ophthalmic complications	E143	E143
Diabetes	Unspecified diabetes mellitus / With neurological complications	E144	E144
Diabetes	Diabetic gangrene		E145
Diabetes	Hyperglycemia hyperosmotic syndrome		E146
Diabetes	Unspecified diabetes mellitus / Without complications	E149	E149
Hypertension	Essential (primary) hypertension	I10-	I10
Hypertension			I110

Hypertension	Hypertensive heart disease without (congestive) heart failure	I119	I119
Hypertension	Hypertensive renal failure		I120
Hypertension	Renovascular hypertension	I150	I150
Hypertension	Renal hypertension		I151
Hypertension	Secondary hypertension		I159
Hyperlipidemia	Pure hypercholesterolemia	E780	E780
Hyperlipidemia	Pure hyperglyceridemia	E781	E781
Hyperlipidemia	Hyperchylomicronemia		E783
Hyperlipidemia	Familial Compound Hyperlipidemia		E784
Hyperlipidemia	Hyperlipidemia, unspecified	E785	E785
Hyperlipidemia	Disorder of lipoprotein metabolism, unspecified	E789	E789
Chronic kidney failure	Chronic kidney disease, stage 1	N181	
Chronic kidney failure	Chronic kidney disease, stage 2		N182
Chronic kidney failure	Chronic kidney disease, stage 3	N183	N183
Chronic kidney failure	Chronic kidney disease, stage 4		N184
Chronic kidney failure	Chronic kidney disease, stage 5	N185	N185
Chronic kidney failure	Chronic kidney disease, unspecified	N189	N189

ICD-10, International Classification of Diseases, Tenth Revision. MDV, Medical Data Vision.
