# Supplementary material for: Analysis of nationwide hemophilia care: A cohort study using two Japanese healthcare claims databases
Source: Health Sci Rep. 2022 Jan 27;5(1):e498. doi: 10.1002/hsr2.498 (PMC8795212; doi:10.1002/hsr2.498)

Supplementary Figure 1. Patients' age distribution in specialized and nonspecialized medical facilities (MDV database).

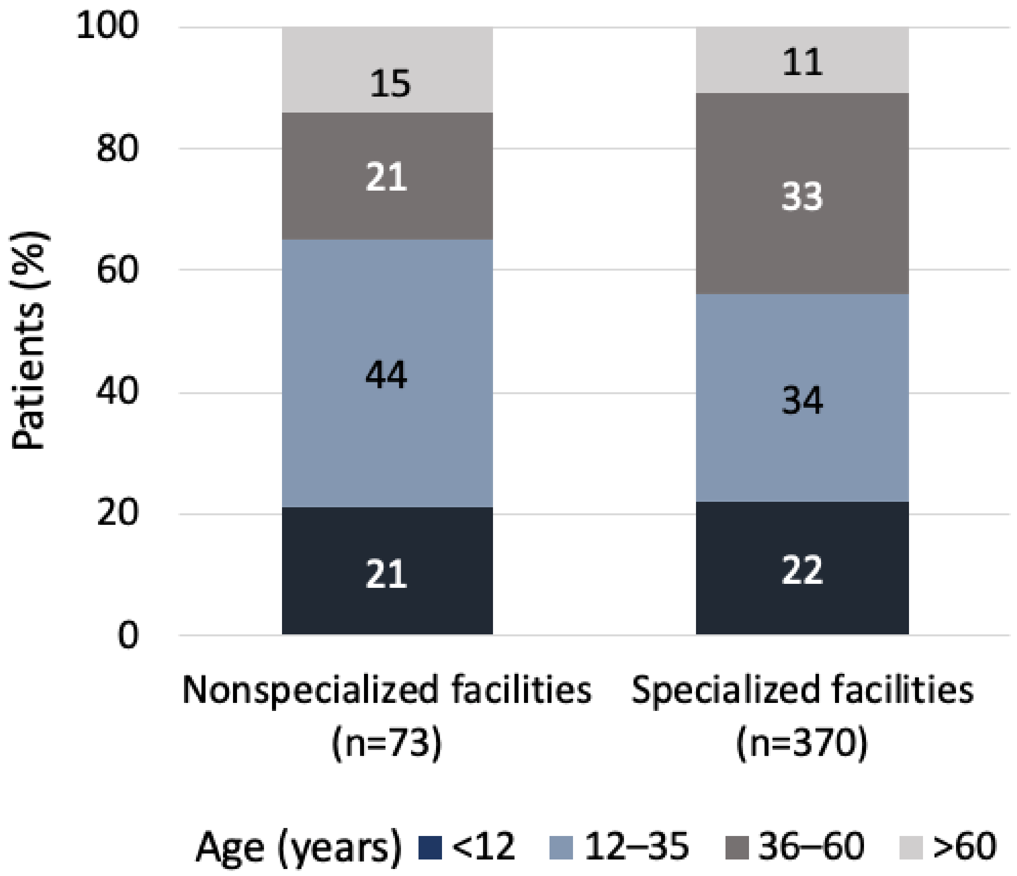

Supplement: Supplementary file 4 — Figure S1. Patients' age distribution in specialized and nonspecialized medical facilities (MDV database). [file HSR2-5-e498-s003.pdf]
